# An electronic pillbox intervention designed to improve medication safety during care transitions: challenges and lessons learned regarding implementation and evaluation

**DOI:** 10.1186/s12913-022-08702-y

**Published:** 2022-10-30

**Authors:** Amrita Shahani, Harry Reyes Nieva, Katie Czado, Evan Shannon, Raquel Gaetani, Marcus Gresham, Jose Cruz Garcia, Hareesh Ganesan, Emily Cerciello, Janan Dave, Rahul Jain, Jeffrey L. Schnipper

**Affiliations:** 1 Pharmacy Department, Brigham and Women’s Hospital, Boston, MA USA; 2 Division of General Internal Medicine and Primary Care, Brigham and Women’s Hospital, 1620 Tremont Street, BC-3, Boston, MA 02120 USA; 3 Department of Medicine, Harvard Medical School, Boston, MA USA; 4 Fellow (Formerly TowerView Health), Philadelphia, PA USA

**Keywords:** Medication safety, Care transitions, Health information technology, Implementation science

## Abstract

**Background:**

Adverse drug events are common during transitions of care. As part of the Smart Pillbox study, a cluster-randomized controlled trial of an electronic pillbox designed to reduce medication discrepancies and improve medication adherence after hospital discharge, we explored barriers to successful implementation and evaluation of this intervention.

**Methods:**

Eligible patients were those admitted to a medicine service of a large teaching hospital with a plan to be discharged home on five or more chronic medications. The intervention consisted of an electronic pillbox with pre-filled weekly blister pack medication trays given to patients prior to discharge. Pillbox features included alarms to take medications, detection of pill removal from each well, alerts to patients or caregivers by phone, email, or text if medications were not taken, and adherence reports accessible by providers. Greater than 20% missed doses for three days in a row triggered outreach from a pharmacist. To identify barriers to implementation and evaluation of the intervention, we reviewed patient exit surveys, including quantitative data on satisfaction and free-text responses regarding their experiences; technical issue logs; and team meeting minutes. Themes were derived by consensus among the study authors and organized using the Consolidated Framework for Implementation Research.

**Results:**

Barriers to implementation included intervention characteristics such as perceived portability issues with the pillbox and time required by pharmacists to enter medication information into the software; external policies such as lack of insurance coverage for early refills and regulatory prohibitions on repackaging medications; implementation climate issues such as the incompatibility between the rushed nature of hospital discharge with the time required to deploy the intervention; and patient issues such as denial of previous problems with medication adherence. We founds several obstacles to conducting the study, including patients declining study enrollment and limited attempts by the hospital to streamline logistics by building the intervention into usual care. Several solutions to address many of these challenges were implemented or planned. Despite these challenges, many patients with the pillbox were pleased with the service and believed the intervention worked well for them.

**Conclusions:**

In this evaluation, several barriers to implementing and conducting a study of the effectiveness of the intervention were identified. Our findings provide lessons learned for others wishing to implement and evaluate HIT-related interventions designed to improve medication safety during care transitions.

**Trial registration:**

Clinicaltrials.gov NCT03475030

## Background

The period following hospital discharge is a vulnerable time for patients who are transitioning home from the acute care setting [1], especially regarding medication use. Patients often have difficulty managing their medications after hospital discharge, due in part to changes in the regimen, challenges in reconciling new medications with what they were taking previously, inadequate discharge instructions, and inadequate follow-up [2]. Unintentional medication discrepancies (i.e., between the prescribed medication regimen and what patients think they should be taking) [3–5] and medication non-adherence (i.e., between what patients think they should be taking and what they actually take) [6, 7] are very common. Such errors in medication use can lead to unnecessary side effects and poor disease control as well as hospital readmission and even death [8–11]. Several studies have shown that post-discharge adverse drug events (ADEs, injury due to a medication in the 30 days after discharge) make up 70% of all post-discharge adverse events, at a rate of 0.30 ADEs per patient; it is estimated that 19% of hospitalized patients suffer an ADE within 30 days of discharge, of which 2/3 are preventable or ameliorable [12–15]. Several studies of interventions designed to reduce post-discharge ADEs, such as pharmacist counseling and follow-up, have had variable success, including several at our own institution [16–18].

Research has found that some health information technology (HIT)-related interventions can enhance adherence and reduce discrepancies [19, 20]. Recent advances include the development of smart medical devices that can track patient outcomes and communicate this information with providers. These devices include “smart pillboxes” that can remind patients to take their medications, track adherence, and send adherence reports to providers [21, 22]. Evaluations of smart pillboxes are emerging in the ambulatory setting but have yet to be deployed or evaluated in the transitions setting, where there are unique logistical challenges, e.g., due to time pressures at discharge, but also tremendous opportunities to engage patients, caregivers, and providers in medication safety and to improve care during a high-risk period in their lives.

The goals of this study were to implement and evaluate a novel “smart pillbox” using HIT, compared to a standard pillbox or no intervention, designed to minimize discrepancies in prescribed regimens and improve adherence after hospital discharge. The purpose of this report is to describe the intervention and our experience with conducting the study, including barriers to implementing and evaluating the intervention and lessons learned for others wishing to deploy complex HIT interventions to improve medication safety during care transitions. Results of the trial on primary outcomes and a qualitative analysis of the impressions of patients, caregivers, and providers using the pillbox will be published in separate manuscripts.

## Methods

### Setting and participants

The Smart Pillbox Transition Study was a cluster-randomized controlled trial, with randomization of patients at the level of the primary care practice. The study was conducted at Brigham and Women’s Hospital (BWH), a 793-bed teaching hospital in Boston, MA, from January 2017 to December 2018. Eligible patients included adults admitted to any inpatient medical service (including general medicine, cardiology, and oncology services) with a BWH primary care provider (PCP), on 5 or more chronic medications, spoke English or Spanish, and had a plan for discharge home. PCPs were typically adult internal medicine trained physicians; the remainder were nurse practitioners. The study was approved by the Partners Healthcare Institutional Review Board and by the BWH Primary Care Practice-Based Research Network.

### Intervention

**Fig. 1:**
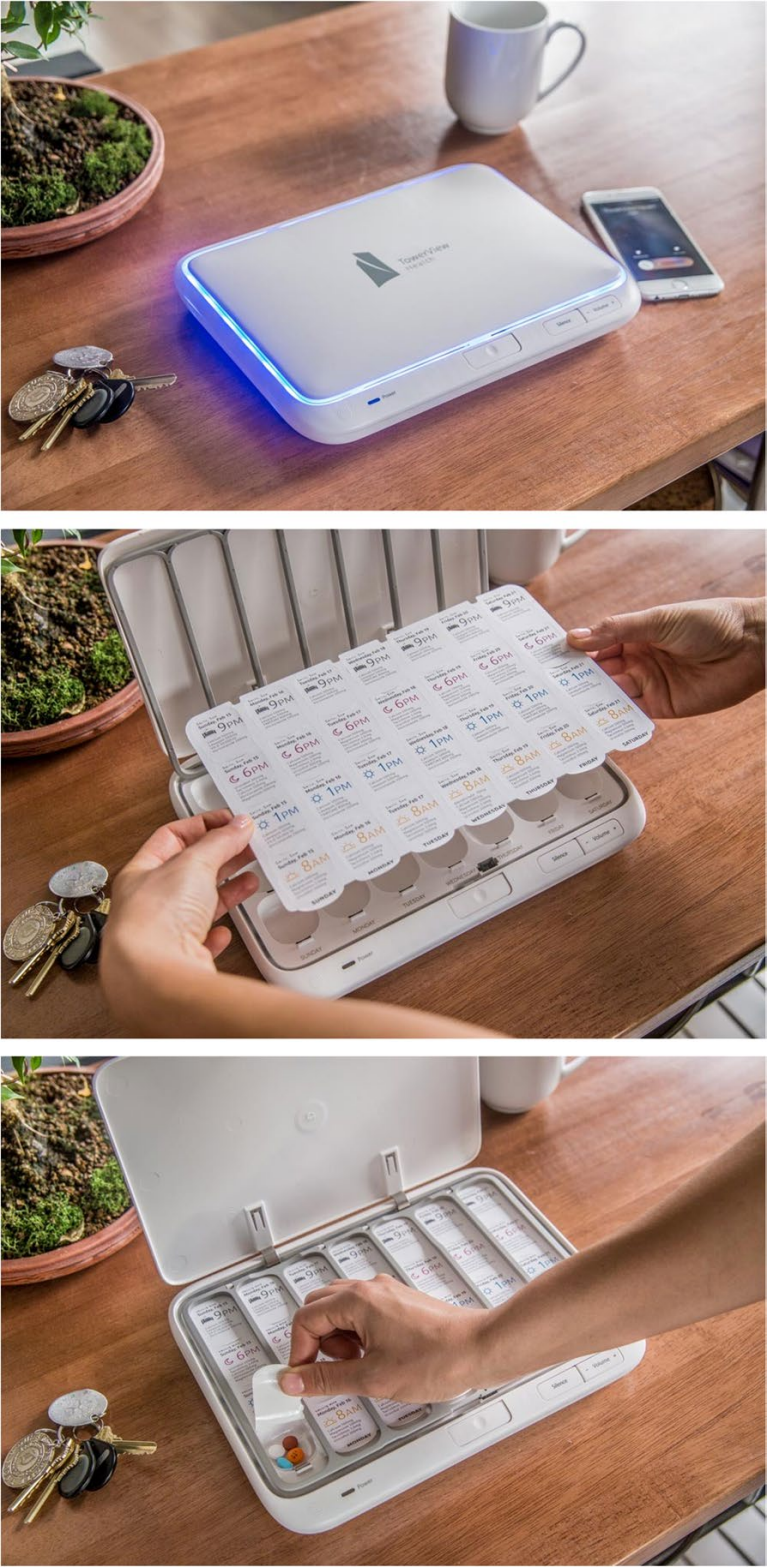
Smart pillbox. Top figure illustrates the closed pill box. Middle figure illustrates insertion of the blister pack into the pill box. Bottom figure illustrates a patient removing pills from one of the wells

The smart pillbox (TowerView, Philadelphia, PA) is a system that accommodates pre-filled weekly medication trays that work in tandem with a connected device that measures real-time medication adherence (Fig. 1). Compartments of the medication trays are heat-sealed with a label that can be peeled back to reveal individual medication compartments for each day of the week (in a Sunday to Saturday format) and time of day. Each compartment is labeled with the name and dosage of the medications it contains. In addition, there is a tear-off card with each tray that contains a detailed description of each medication and directions for use. Non-oral medications (e.g., inhalers, nasal sprays, injectables), frequently changing medications (e.g., diuretics, warfarin), as-needed (PRN) medications, and opioids and other controlled substances were not included in the medication trays.

**Fig. 2:**
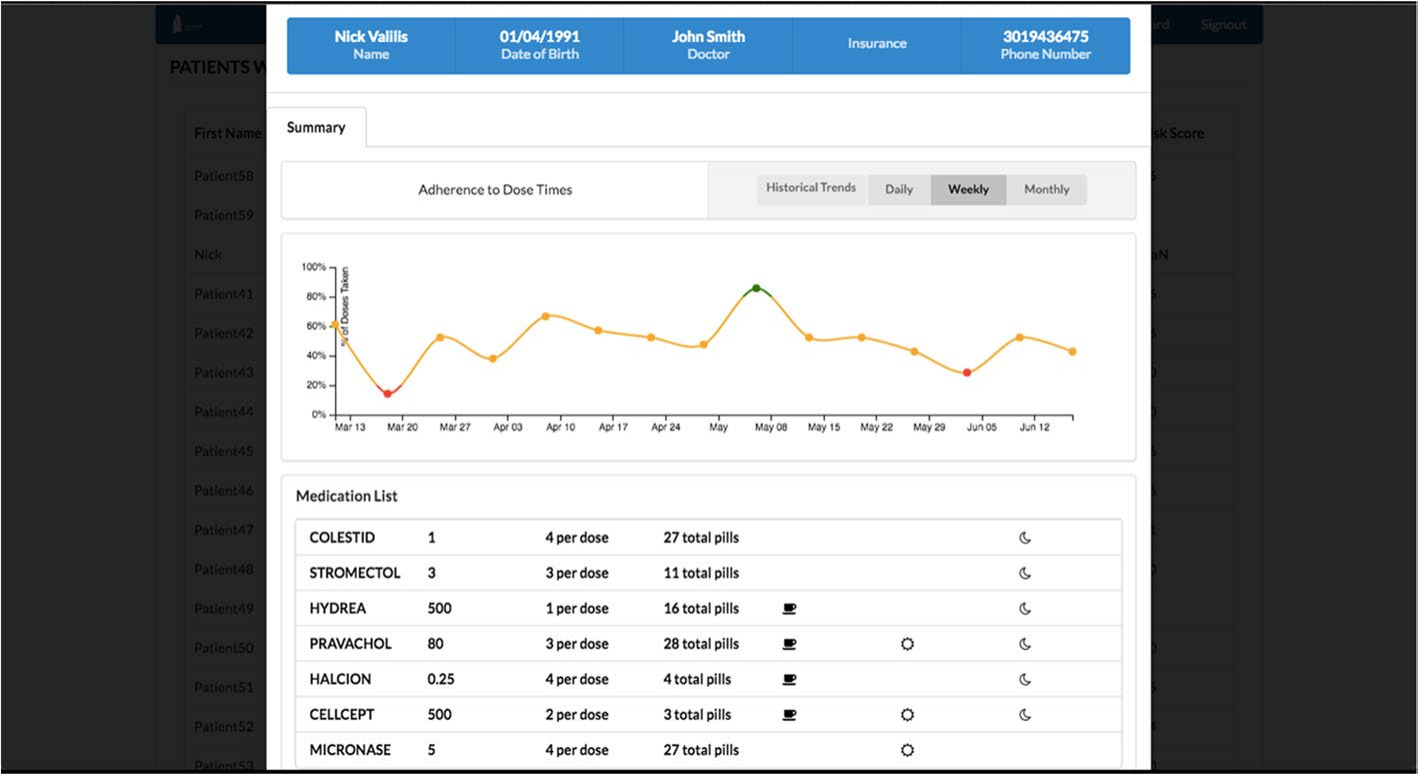
Medication Adherence Reports. Top figure illustrates a sample adherence report (trend graph) by week. Bottom graph illustrates a “heat map” with number of doses removed and mean timing of removal for each administration day and time over the course of one month

Once the medication regimen and times of day for medication usage are entered into the pillbox’s secure online application, and the medication labels are printed and applied to the filled medication tray, the pillbox is ready for use. When it is time for a patient to take their medications, an alarm chimes and the appropriate compartment of the tray lights up. The pillbox optically senses if all the medications have been removed from the compartment. If not, the alarm chimes again and a text message, phone, or email reminder can be sent to the patient and/or a designated caregiver. Information regarding whether medications were removed from each compartment and the time of removal are stored and then transmitted on a cellular network (Verizon) to a central server. The data are aggregated into an adherence report by week and by month. The list of patients for each PCP is prioritized by those patients with low adherence (> 20% missed doses in the prior 3 days) at the top. Clicking on a patient’s name displays adherence rates (proportion of doses taken) over time. A second view, known as the “heatmap,” shows which doses have been missed and how many minutes late each dose was taken compared to the prescribed time. These data can be viewed for any week or aggregated monthly to produce summaries by date and time (e.g., 2 of 4 doses taken on Friday afternoons, average of 14 min late; Fig. 2).

The initial medication tray was created by the BWH ambulatory pharmacy (see below for enrollment process), while subsequent medication trays were filled either by the BWH ambulatory pharmacy or by a dedicated third-party pharmacy (Curant Health, Smyrna, GA), depending on the patient’s insurance. These trays were mailed to the patient every 2–4 weeks. Prior to filling these trays, the pharmacy would review the medication regimen according to the patient’s electronic medical record (Epic, Verona, WI) to identify any discrepancies with their list of prescriptions. A pharmacist would also contact the patient to confirm the regimen. Any discrepancies were then resolved by contacting the patient’s PCP or other prescribers as needed.

A pharmacist at the filling pharmacy also reviewed each patient’s medication adherence report on a weekly basis. In the event of > 20% missed doses for 3 days in a row, the pharmacist would call the patient to discuss possible barriers to medication adherence and create a plan to overcome these barriers. Documentation of these actions were added to each patient’s electronic medical record. We also created a link to the adherence report within the EHR's environment and provided access to each patient’s PCP. PCPs (or their practice managers) were encouraged to periodically review these reports and to take action as they saw fit.

### Patient enrollment

Prior to the start of the study, researchers met with each BWH primary care practice to explain the intervention, the study, and their role in it, and were given the option to participate or not. We also addressed any questions (e.g., what to do if they change the medication regimen after discharge). Practices that chose to participate were then randomized to the smart pillbox, simple pillbox, or usual care.

Patients who met study criteria were identified shortly after admission by a research assistant using a report generated by our EHR. Once eligibility criteria were confirmed by targeted medical record review and discussion with the inpatient medical team, patients were asked to provide informed written consent and to complete a brief intake questionnaire. Once enrolled, patients’ PCPs and inpatient providers were contacted informing them of study enrollment and were given an opportunity to opt the patient out of the study prior to discharge if they felt the patient was a poor candidate for the intervention. Allocation was concealed from patients until after the consent process was complete. The study duration was 6 months from the time of discharge.

### Usual care and simple pillbox arms

Patients in the usual care arm had their medication prescriptions electronically transmitted to the community pharmacy of their choice or to the BWH ambulatory pharmacy if they preferred. Medical teams could still opt for medication bedside delivery by the BWH ambulatory pharmacy if desired, although this was not common. All patients in the study, regardless of arm, received counseling on their discharge medication regimen from their nurse or a unit-based pharmacist. In addition, most patients admitted to medicine through the Emergency Department had a “best possible medication history” taken by a trained pharmacy technician or resident, who documented that history in Epic so that it could be used for discharge medication reconciliation. Lastly, the medication reconciliation screens in Epic were previously optimized to maximize the clarity of the medication orders at hospital discharge.

Patients assigned to the simple pillbox were given a weekly pillbox on the day of discharge and provided instructions on how to fill it, including a manually created table of their medications, the quantity to take at each time of day, and other directions for how to use the pillbox on a daily and weekly basis.

### Smart pillbox deployment

For patients assigned to the intervention, the research assistant (RA) contacted their responding clinician (usually an intern or physician assistant) by email and then page, notifying them of the patient’s participation and providing instructions about their role in the study: 1) to perform discharge medication reconciliation and send prescriptions to the BWH ambulatory pharmacy as early in the discharge process as possible to allow sufficient time for the pharmacy to create the medication trays; 2) complete an online bedside medication delivery form, including the estimated date and time of discharge, a note of any anticipated last-minute changes to the discharge medication regimen, and a note of any medications that should be withheld from the pillbox due to unexpected future changes to the regimen (e.g., furosemide in a patient with unstable congestive heart failure).

RAs contacted the inpatient units caring for participants each weekday to identify those being discharged that day. Based on the patient’s discharge regimen and their insurance, the patient’s total medication copayments were determined and then conveyed to the patient. Because copayments could in theory increase, e.g., if 90-day mail-order prescriptions of $20 were changed to three 30-day prescriptions of $10 each, patients were given the option to withdraw from the study at that time (contractually in the US, patients are expected to pay their copayments, and the pharmacy cannot routinely waive copayments). Because medications cannot be repackaged (i.e., in a medication tray) by a pharmacist by federal and state regulations, any medication recently filled prior to admission and to be prescribed at discharge could be declined by insurance as an early refill. We therefore set up a fund as part of the grant to reimburse the pharmacy for any uncovered medications.

On the day of discharge, a trained BWH pharmacist contacted each patient, verified the times of day the patient planned to take each medication, and elicited information about any caregivers to be contacted in the event of missed/late medication doses. The pharmacist entered this information in the pillbox software and created two full weeks of medication trays plus a short week to account for the day of discharge (e.g., if the patient was discharged on a Thursday, the first tray would contain Thursday through Saturday medications, with two more trays for medications Sunday through Saturday). Since the Epic EHR and the pillbox software were not compatible with each other, we programmed the EHR to create a CSV file of the discharge medication regimen that could be imported into the pillbox software to facilitate this process. However, the study pharmacist still needed to manually verify the accuracy of all imported information, and manual entry of certain fields, such as pill identification number, was still necessary.

Immediately prior to discharge, the pharmacist delivered the pillbox and the medication trays to the patient’s bedside, demonstrated how to use the intervention, and answered any questions. Patients were also provided with a Frequently Asked Questions brochure and Let’s Get Started booklet. Patients were provided with a number to call for any technical or medication-related issues (staffed by Curant or BWH ambulatory pharmacists working on the study). Finally, the patient’s pharmacy was updated in the EHR.

For patients discharged on weekends, when the ambulatory pharmacy was closed, plans were made for patients to be given a 3–4-day supply of medications in pill bottles (from the BWH ambulatory pharmacy on the Friday prior to discharge) and for them to return to the BWH ambulatory pharmacy to pick up their pillbox and receive instructions on its use on Monday.

Patients continued to use the pillbox for up to 6 months after discharge. At that point, participation in the study ended, and patients were given the option to go back to their former way of managing medications with their prior pharmacy, to continue using the medication trays without the pillbox, or with permission from TowerView, to continue to use the intervention.

### Data collection

To better understand challenges to implementing and evaluating the intervention, we collected data from a variety of sources: 1) relevant minutes from weekly research team meetings, which included research personnel from BWH and operational personnel from TowerView; 2) logs of technical issues with the pillbox sent to TowerView by patients and caregivers; and 3) patient satisfaction surveys administered by phone to intervention patients at the end of the 6-month follow-up period. The surveys included both quantitative satisfaction questions and free response questions regarding their general reactions and experiences. Together, these sources provided a view of implementation challenges from all stakeholders: patients and caregivers interacting with the intervention; study pharmacists involved in deploying the intervention; TowerView and Curant staff involved in intervention design, deployment, and technical support; and research personnel involved in subject enrollment, project management, and data collection. Themes were generated from this information and reviewed with all study authors until consensus was reached, using an inductive approach, i.e., based on findings evident in the raw data. We divided themes into those related to the implementation of the pillbox and those related to conducting the research study. *Post-hoc*, we used the Consolidated Framework for Implementation Research (CFIR) [23] to categorize barriers to implementation. CFIR provides a menu of 26 constructs, grouped into 5 domains, that have been associated with effective implementation based on prior studies of implementation science. Depending on the situation, each construct (e.g., an individual’s state of change) could be a barrier or facilitator of implementation. In the Results, we present domains in **boldface** and underline (and define) constructs.

## Results

**Fig. 3:**
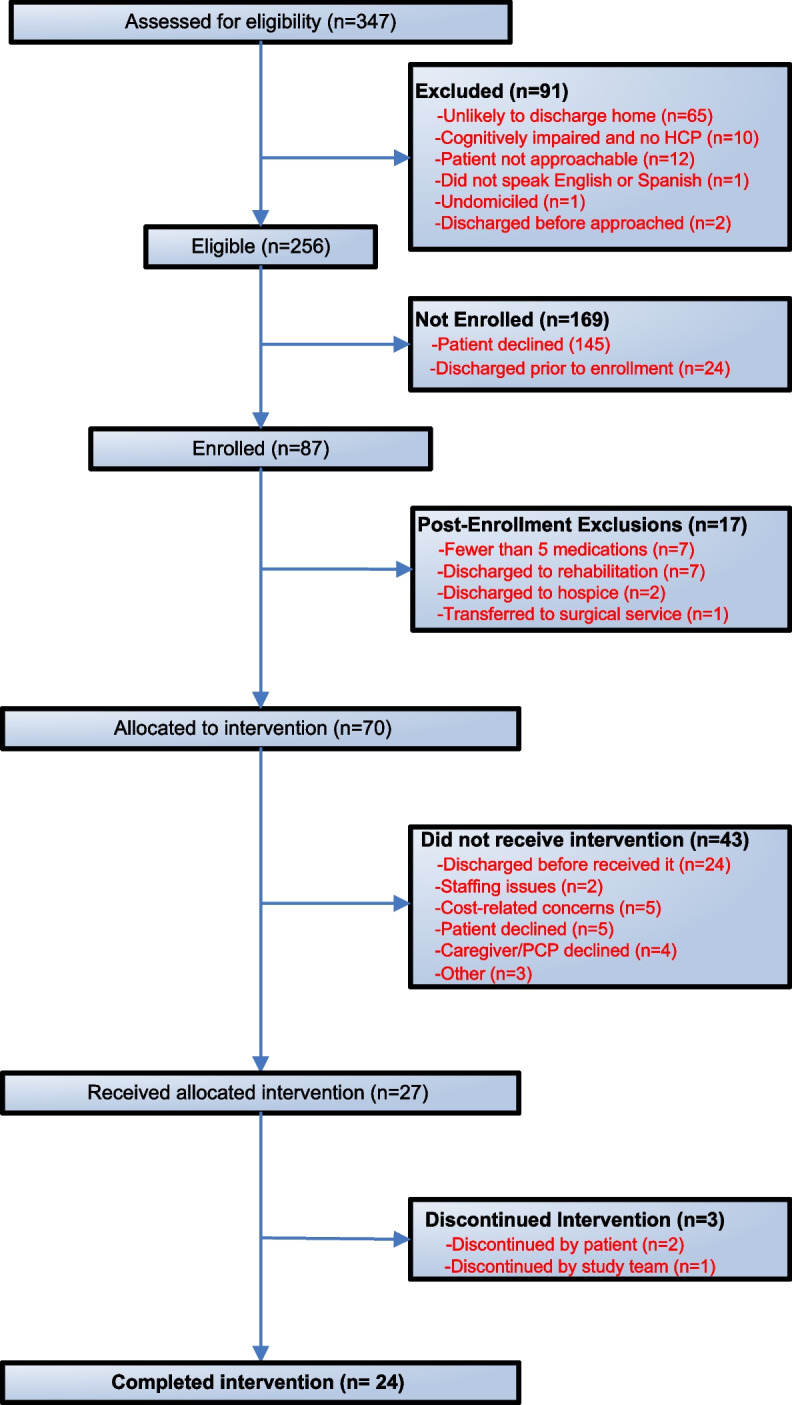
Flow diagram of intervention patients. Abbreviations: HCP: healthcare proxy; PCP: primary care provider

The flow diagram of patients assigned to the intervention arm is shown in Fig. 3. Of 256 eligible patients, 169 were not enrolled, mainly because patients declined to participate. Of the 87 patients who were enrolled, 63 did not complete the intervention. This was for a variety of reasons, but the most common was that the patient was discharged before the pillbox could be provided and they chose not to return to pick it up after discharge. In several cases, patients no longer met eligibility criteria (e.g., discharged to hospice or rehabilitation, on fewer than five medications). In other cases, patients withdrew from the study, or their PCP (or study staff) opted them out of the study after enrollment. The cost of copayments was the specified reason for patient withdrawal in 5 cases. Regarding use of the intervention by primary care, PCPs and practice managers rarely accessed the adherence reports during the study period. Because of low enrollment, we decided, in conjunction with the Agency for Healthcare Research and Quality (AHRQ, the study’s funder), to eliminate the simple pillbox arm of the study and to focus efforts on the main comparison between the full intervention and usual care.

### Barriers to intervention implementation

Barriers to implementation of the intervention could be divided into several categories, corresponding to CFIR constructs (Table 1). These barriers were derived predominantly from weekly meeting minutes (nearly every meeting raised at least one of these barriers), complemented with approximately two dozen logs of technical issues and free-response answers to the patient satisfaction surveys (17 of the 24 patients who completed the intervention completed these surveys).

**Table 1:** Barriers to implementation and potential solutions

**CFIR Domain**	**CFIR Construct**	**Specific Element**	**Potential Solutions**	**Status of Solution**
Intervention Characteristics	Adaptability	Perceived portability issues with pillbox	Educate patient that pills may be removed early in the day (i.e., before going out)	Adopted, with moderate success
		Pillbox connectivity: poor signal in some locations	Planned pillbox enhancements	Not adopted during trial
			Optimize location of the pillbox within the home	Adopted, need to balance with accessibility of box, ability to hear alarms
		Pillbox threshold for detecting removal of small pills	Group medications for each dose if possible	Adopted, with moderate success
			Planned pillbox enhancements to detect one small pill	Not adopted during trial
	Complexity	Time required to dispense medications and enter information into pillbox application	Develop pillbox software interface compatible with hospital EHR system	Not adopted during trial
Outer Setting	External Policies and Incentives	Lack of insurance coverage for early prescription refills	Plan to engage insurance companies to allow for early refills	Not adopted during trial
			Grant paid for recently dispensed medications	Adopted, successful (but not sustainable)
		Regulations that prohibit pharmacists from repackaging previously dispensed medications	Plan to work with state agencies re: whether filling a pillbox counts as repackaging	Not adopted during trial
		Potential for copayments to increase for monthly fills	Emphasize to patients that benefits of the intervention may be worth the copay increase	Adopted, with limited success
			Engage insurance companies to waive co-payment increase for smart pillboxes	Not adopted during trial
Inner Setting	Implementation Climate: Compatibility	Too many medications dispensed outside of the pillbox	Text reminders for non-pillbox medications	Adopted, with moderate success
			Patient education re: using pillbox under different situations	Adopted, with moderate success
		Turn-around time: pharmacy often received prescriptions < 2 h before anticipated discharge	Encouraged clinicians to provide prescriptions as early as possible	Adopted, with limited success
			Facilitated early communication between pharmacist and clinician	Adopted, with limited success
		Outpatient pharmacy closed on weekends	Developed protocol for patients discharged over the weekend to return on Monday to receive pillbox	Adopted, with limited success (patients often did not come back)
		Difficulty obtaining prescription refills from providers, esp. if multiple prescribers per patient	Procedures for obtaining refills from each practice and documenting usual prescriber for each medication	Adopted, with moderate success
Characteristics of Individuals	Individual state of change	Patient denial of previous problems with adherence	Scripts to reduce stigma of accepting the intervention	Adopted, with limited success
			Engagement of patient’s caregivers and providers	Adopted, with limited success
	Other personal attributes	Difficulty reaching patients to confirm refills	Attempt to reach patients through multiple methods in addition to phone calls	Adopted, with limited success

Some barriers were attributable to the **intervention** itself, especially its complexity (perceived difficulty of the intervention, reflected by scope, disruptiveness, number of steps, etc.) and adaptability (the degree to which an intervention can be adapted, tailored, refined, or reinvented to meet local needs), e.g., the time required to enter medications into the pillbox application due to lack of compatibility with the hospital’s EHR. Technical issues included poor signal in some locations, leading to failure to record adherence data, and the occasional inability for the optical reader to detect the removal of medications, e.g., if only one small pill was in the compartment.

Other barriers were due to the **outer setting**, including external policies and incentives (external factors that affect spread, including governmental regulations, guidelines, pay-for-performance, public reporting, etc.), such as issues with insurance coverage of early refills and regulatory prohibitions on pharmacist repackaging of medications, as noted earlier. Another common type of barrier was the **inner setting**, implementation climate (local capacity for change, shared receptivity to an intervention, extent to which use of an intervention is rewarded, supported, and expected within an organization), including the sub-construct of *compatibility* (degree of tangible fit between meaning and values attached to the intervention by involved individuals vs. perceived risks and needs, and how well the intervention fits with existing workflows and systems), such as the lack of compatibility between the rushed and unpredictable nature of hospital discharge with the time required to prepare the pillbox. In some cases, patients had too many medications dispensed outside the pillbox (e.g., non-oral medications, controlled substances, as needed medications, medications at risk for frequent changes) which made them a poor fit for the intervention. Also, the BWH ambulatory pharmacy was closed on weekends, thus creating a separate workflow for patients discharged over the weekend, a process that did not always work. Finally, there were **patient** barriers, including their individual state of change (phase the individual is in (e.g., precontemplation, contemplation) as they progress toward skilled, enthusiastic, and sustained use of the intervention), such as their denial of problems with adherence and therefore their reluctance to accept help. Other patients were simply overwhelmed by their hospitalization and did not want any additional changes to their routine. We also encountered difficulty reaching patients after discharge to confirm regimens prior to mailing new medication trays.

### Barriers to conducting the research

We also encountered several barriers to conducting the research itself. For example, some patients were simply resistant to participating in research studies. In addition, as a research study, the intervention was by definition not built into usual care, so attempts to streamline logistics at the hospital level (e.g., solving compatibility issues between the pillbox’s software and the hospital pharmacy’s dispensing software) were limited.

### Solutions to barriers

Several solutions were implemented during the course of the study to mitigate the barriers that came up during the study, while others were discussed, not adopted during the trial, and recommended for future implementation, both at our facility and at other facilities looking to introduce similar interventions (Table 1). For example, we revised our enrollment scripts several times to reduce the perceived stigma of participating in the study, and when patients were unsure about enrolling, we enlisted the help of the patient’s inpatient and outpatient providers and their caregivers to gently encourage participation if appropriate. We worked with the pillbox vendor to create a system of text reminders for medications outside the pillbox to remove the disparity between those medications that could and could not leverage the intervention. We made several changes to our protocol to improve communication between study staff and the inpatient medical team to facilitate the completion of pre-discharge tasks. We also improved our procedures to identify the correct prescriber for each outpatient medication in order to obtain prescription renewals as efficiently as possible. Some of these efforts were more successful than others (Table 1). Planned improvements that did not occur during the trial included pillbox software interfaces compatible with the hospital’s EHR and medication dispensing software, and technical improvements regarding signal strength and optical detection of pill removal.

Despite these obstacles, exit surveys with patients who used the pillbox were extremely positive. They often noted the convenience of their medications being mailed to them, increased confidence knowing it was the correct regimen, and no longer having the burden (or their family’s burden) to fill their own pillbox or use pill bottles to organize their regimens. Opinions were mixed on the reminder system, but some patients found it helpful.

## Discussion

In summary, we identified several obstacles to enrolling patients in this study and successfully providing intervention patients with the smart pillbox prior to hospital discharge. The obstacles to implementation corresponded to known CFIR domains, including the intervention itself (e.g., lack of portability of the pillbox), outer setting (e.g., regulations against repackaging of medications), inner setting (e.g., lack of compatibility in workflows for discharge and preparing the pillbox), and patients (e.g., denial of need for the intervention). Those who used the intervention were pleased with it, but obviously there is selection bias among those who chose to go through the obstacles to use it and liked it enough to continue to use it.

There are several likely explanations for our findings. Discharge planning can be a complicated process involving much logistical planning, such that adding a process such as a smart pillbox in a short amount of time can create many unforeseen barriers (i.e., lack of compatibility of workflows). The intervention required approximately 2 h from the time prescriptions were received to when the pillbox was filled and delivered. Tasks included obtaining pharmacy benefits information, processing prescriptions, troubleshooting insurance issues, entering medication information into the TowerView software platform, printing patient specific labels, filling the pillbox, and coordinating delivery and education to the patient. Often, prescriptions were sent to the pharmacy less than 2 h prior to the expected time of discharge, resulting in a rush to provide the intervention and sometimes in an inability to provide it at all. Competing priorities, such as reducing length of stay or discharging patients before noon, also complicated these logistics.

One of the major barriers faced by the pharmacy was using the software for inputting the information into the TowerView platform (intervention complexity). There was no interface between the Towerview platform and either the hospital’s EHR or the pharmacy’s medication dispensing software. While a CSV file of the discharge medication regimen could be created by the EHR and then uploaded to the TowerView system, it still required double-checking to make sure all the information was accurate. And as noted above, medication information such as pill identification number had to be manually entered, unlike usual care. These were extremely time-consuming steps and the biggest barrier from the pharmacy for a timely turnaround and providing this as a long-term service.

This study complements and adds to what is already known about the barriers to implementing HIT interventions. For example, two recent reviews used CFIR to better understand barriers and facilitators of HIT implementation, one regarding learning health systems [24] and the other regarding chronic disease management [25]. The most commonly cited factors included Intervention constructs such as Relative Advantage over other interventions, Inner Setting constructs such as Culture, Implementation Climate (including compatibility with workflow), Readiness for Implementation (including leadership engagement and available resources), and Individual Characteristics such as health literacy and digital literacy. Our study corroborates the importance of compatibility with workflow while emphasizing other constructs such as Adaptability and Complexity of the intervention and External Policies and Incentives. This study also adds to what is known, mostly from the field of pragmatic clinical trials, on the challenges of doing research in complex, dynamic, real-world, clinical settings, and the need for early and ongoing engagement with health system leaders and front-line clinicians [26], as well as with patients (e.g., through patient-family advisory councils) [27].

There are several implications of our findings. Given the known problems with medication safety after hospital discharge and the potential of interventions like this to address these problems, the larger questions are whether this intervention should become part of usual care, and if so, what it would take for this kind of intervention to become part of usual care. The answer to the first question requires the results of the cluster RCT, which is pending. Regarding the second question: if no longer part of a research study, several obstacles (such as the need for consent or patient resistance to participating in research) would be resolved. However, issues of patient denial of prior medication problems (i.e., individual characteristics: state of change) may persist: we frequently noted feelings of shame and guilt around prior medication-taking behavior, and offering this intervention as an optional part of usual care may not solve this problem, even if offered by an established provider (or care coordinator) as opposed to research staff. This is not that different from offering other services to help patients, such as health coaches, where it is paramount to minimize the stigma associated with accepting help.

Logistical issues were prominent barriers (manifested as intervention complexity and lack of compatibility of workflows), and some could be resolved by “productizing” this intervention. For example, ensuring software compatibility of pillbox software and the EHR, having multiple pharmacists trained in programming the software and dispensing blister packs, and taking advantage of economies of scale would likely help a great deal. There would also need to be a more concerted effort to facilitate early communication between prescribing clinicians and pharmacists, especially around early provision of discharge prescriptions. Nevertheless, it is likely that implementing this intervention would always take longer than not implementing it, and so the costs and benefits would need to be more clearly defined to make the case for using it, including the opportunity costs of hospital pharmacists (i.e., what else they could be doing with their time). Moreover, some logistical issues are harder to correct, such as the tension between time constraints to set up a pillbox and the rush and unpredictability of hospital discharge, and the restricted hours of most hospital-based ambulatory pharmacies, which make evening and weekend discharges challenging. However, these may be solvable through continuous engagement and co-design with stakeholders. It may also be the case that some of the benefits would still accrue to patients by initiating the intervention well after discharge, eliminating the rush to deploy the intervention prior to them leaving the hospital.

Some issues would require more systemic change. For example, US insurance companies could agree to a waiver of early refills and a reduction in copayments to 90-day levels in exchange for using the intervention. They may find that the improvements in medication adherence and disease control, possibly leading to reduced health care utilization, are worth it. Another question is whether there is a sustainable business model for pharmacies to do the extra work, and if not, who pays for it. The business case might be clearest for self-insured integrated delivery systems (or in countries with single-payor health systems), where investments in time and resources may be practical in exchange for reduced downstream costs. For example, a pharmacist-led medication therapy management program reduced health care costs and led to a positive return on investment in one self-insured plan [28].

Lastly, some issues require iterative technological improvements, including signal strength, pillbox connectivity, thresholds for detecting pill removal, and improving portability. We should also acknowledge that some patients may not be ideal candidates for this intervention, including those with many medications outside the pillbox or with frequently changing regimens. Patients who travel frequently were also resistant to using the pillbox. Patients who were not technically savvy or with mild cognitive impairment were resistant to using the pillbox even though they may be the ones to benefit the most, especially with assistance from a family caregiver. There may also be a point at which cognitive impairment is too severe for this intervention to work.

Several technologies to improve medication organization and adherence are beginning to emerge, each with its own advantages and disadvantages. The smart pillbox used in this study, with pharmacist-provided prefilled medication trays, is likely effective at reducing medication discrepancies, especially if the regimen is reviewed with the EHR prior to each new shipment. However, it has disadvantages in terms of portability and issues with frequently changing regimens. Other technologies, such as the Philips automated medication dispensing device (Philips NA, Cambridge, MA) [29] and MedaCube (PharmAdva, Rochester, NY), are even less portable but more able to accommodate frequent regimen changes. At the other end of the spectrum are individually wrapped medication pouches for each dose, mailed to the patient’s home (such as PillPack, Manchester, NH), which are more portable but less accommodating to medication changes and do not promote or detect medication adherence. In general, most of these technologies are not well-studied, although there are a few studies evaluating other smart pillboxes [22, 30, 31]. To our knowledge, none have been studied in the transitional care setting.

## Conclusions

In conclusion, we identified several barriers with implementing and evaluating a smart pillbox designed to decrease medication discrepancies and improve medication adherence during care transitions. The lessons learned in this report are likely to be instructive for others interested in implementing and studying HIT-related interventions at the time of hospital discharge to improve medication safety. These lessons include the need to maximize adaptability and minimize complexity of the intervention (e.g., by ensuring software compatibility), manage the tension between intervention deployment and the rushed and unpredictable nature of hospital discharge, build the intervention into usual care to improve efficiency and economies of scale, and anticipate regulatory and financial issues and patient resistance to accepting help. The challenges to conducting research in complex and dynamic environments requires continuous engagement of stakeholders, including healthcare leadership, front-line clinicians, and patients.

## Data Availability

The datasets used and/or analyzed during the current study are available from the corresponding author on reasonable request.

## References

[1] Coleman EA, Berenson RA (2004). Lost in transition: challenges and opportunities for improving the quality of transitional care. Ann Intern Med.

[2] Pippins JR, Gandhi TK, Hamann C (2008). Classifying and predicting errors of inpatient medication reconciliation. J Gen Intern Med.

[3] Boockvar KS, Liu S, Goldstein N, Nebeker J, Siu A, Fried T (2009). Prescribing discrepancies likely to cause adverse drug events after patient transfer. Qual Saf Health Care.

[4] Climente-Marti M, Garcia-Manon ER, Artero-Mora A, Jimenez-Torres NV (2010). Potential risk of medication discrepancies and reconciliation errors at admission and discharge from an inpatient medical service. Ann Pharmacother.

[5] Coleman EA, Smith JD, Raha D, Min SJ (2005). Posthospital medication discrepancies: prevalence and contributing factors. Arch Intern Med.

[6] Cohen MJ, Shaykevich S, Cawthon C, Kripalani S, Paasche-Orlow MK, Schnipper JL (2012). Predictors of medication adherence postdischarge: the impact of patient age, insurance status, and prior adherence. J Hosp Med.

[7] Cua YM, Kripalani S (2008). Medication use in the transition from hospital to home. Ann Acad Med Singapore.

[8] Gehi AK, Ali S, Na B, Whooley MA (2007). Self-reported medication adherence and cardiovascular events in patients with stable coronary heart disease: the heart and soul study. Arch Intern Med.

[9] Ho PM, Rumsfeld JS, Masoudi FA (2006). Effect of medication nonadherence on hospitalization and mortality among patients with diabetes mellitus. Arch Intern Med.

[10] Ho PM, Spertus JA, Masoudi FA (2006). Impact of medication therapy discontinuation on mortality after myocardial infarction. Arch Intern Med.

[11] McDermott MM, Schmitt B, Wallner E (1997). Impact of medication nonadherence on coronary heart disease outcomes. A critical review. Arch Intern Med.

[12] Forster AJ, Clark HD, Menard A (2004). Adverse events among medical patients after discharge from hospital. CMAJ.

[13] Forster AJ, Murff HJ, Peterson JF, Gandhi TK, Bates DW (2003). The incidence and severity of adverse events affecting patients after discharge from the hospital. Ann Intern Med.

[14] Forster AJ, Murff HJ, Peterson JF, Gandhi TK, Bates DW (2005). Adverse drug events occurring following hospital discharge. J Gen Intern Med.

[15] Tsilimingras D, Schnipper J, Duke A (2015). Post-discharge adverse events among urban and rural patients of an urban community hospital: a prospective cohort study. J Gen Intern Med.

[16] Kripalani S, Roumie CL, Dalal AK (2012). Effect of a pharmacist intervention on clinically important medication errors after hospital discharge: a randomized trial. Ann Intern Med.

[17] Kucukarslan SN, Peters M, Mlynarek M, Nafziger DA (2003). Pharmacists on rounding teams reduce preventable adverse drug events in hospital general medicine units. Arch Intern Med.

[18] Schnipper JL, Kirwin JL, Cotugno MC (2006). Role of pharmacist counseling in preventing adverse drug events after hospitalization. Arch Intern Med.

[19] Marien S, Krug B, Spinewine A (2017). Electronic tools to support medication reconciliation: a systematic review. J Am Med Inform Assoc.

[20] Mistry N, Keepanasseril A, Wilczynski NL (2015). Technology-mediated interventions for enhancing medication adherence. J Am Med Inform Assoc.

[21] Choi EPH (2019). A pilot study to evaluate the acceptability of using a smart pillbox to enhance medication adherence among primary care patients. Int J Environ Res Public Health.

[22] Spratt ES, Papa CE, Mueller M (2017). Using technology to improve adherence to HIV medications in transitional age youth: research reviewed, methods tried, lessons learned. J Gen Med (Dover).

[23] CFIR Research Team. Center for Clinical Management Research. Consolidated Framework for Implementation Research. 2022. Retrieved July 1, 2022, from https://cfirguide.org/.

[24] Rajamani S, Hultman G, Bakker C, Melton GB (2022). The role of organizational culture in health information technology implementations: a scoping review. Learn Health Syst.

[25] Sung M, He J, Zhou Q (2022). Using an integrated framework to investigate the facilitators and barriers of health information technology implementation in noncommunicable disease management: systematic review. J Med Internet Res.

[26] Weinfurt KP, Hernandez AF, Coronado GD (2017). Pragmatic clinical trials embedded in healthcare systems: generalizable lessons from the NIH Collaboratory. BMC Med Res Methodol.

[27] Harrison JD, Auerbach AD, Anderson W (2019). Patient stakeholder engagement in research: a narrative review to describe foundational principles and best practice activities. Health Expect.

[28] Wittayanukorn S, Westrick SC, Hansen RA (2013). Evaluation of medication therapy management services for patients with cardiovascular disease in a self-insured employer health plan. J Manag Care Pharm.

[29] Reeder B, Demiris G, Marek KD (2013). Older adults' satisfaction with a medication dispensing device in home care. Inform Health Soc Care.

[30] Dunn KE, Brooner RK, Stoller KB (2021). Technology-assisted methadone take-home dosing for dispensing methadone to persons with opioid use disorder during the Covid-19 pandemic. J Subst Abuse Treat.

[31] Vaz KKH, Carmody JK, Zhang Y, Denson LA, Hommel KA (2019). Evaluation of a Novel Educational Tool in Adolescents With Inflammatory Bowel Disease: The NEAT Study. J Pediatr Gastroenterol Nutr.

